# Artificial Intelligence‐Based In Silico Evaluation of the Pharmacological Potential and In Vitro Anti‐Malignancy Effect in the Human Glioblastoma Cell Line of the Hydrate of the Coumarin Compound Meranzin Originating From the Edible Macroalga (*Bangia fuscopurpurea*)

**DOI:** 10.1002/fsn3.71332

**Published:** 2025-12-29

**Authors:** Shi‐Ying Huang, Yi‐Chen Chang, Charles Chien‐Chih Chiu, Chang‐Wei Hsieh, Chien‐Wei Feng, Zhi‐Cheng Chen, Nan‐Fu Chen, Yung‐Kuo Lee, Tian‐Huei Chu, Cheng‐Chieh Fang, Nan‐Chieh Huang

**Affiliations:** ^1^ College of Ocean Food and Biological Engineering Jimei University Xiamen China; ^2^ Doctoral Degree Program in Marine Biotechnology National Sun Yat‐Sen University and Academia Sinica Kaohsiung Taiwan; ^3^ Department of Biotechnology Kaohsiung Medical University Kaohsiung Taiwan; ^4^ Department of Food Science and Biotechnology National Chung Hsing University Taichung Taiwan; ^5^ Department of Food Science National Ilan University Yilan Taiwan; ^6^ Department of Medical Research China Medical University Hospital Taichung Taiwan; ^7^ Department of Obstetrics and Gynecology Kaohsiung Medical University Hospital, Kaohsiung Medical University Kaohsiung Taiwan; ^8^ Center for Cancer Research Kaohsiung Medical University Kaohsiung Taiwan; ^9^ Tongde Traditional Chinese Medicine Clinic Taoyuan Taiwan; ^10^ Institute of Medical Science and Technology National Sun Yat‐Sen University Kaohsiung Taiwan; ^11^ Center for Neuroscience National Sun Yat‐Sen University Kaohsiung Taiwan; ^12^ Department of Information Management I‐Shou University Kaohsiung Taiwan

**Keywords:** cytotoxicity, human glioblastoma cell lines, in silico evaluation, invasion inhibition, sphere formation inhibition

## Abstract

In 2023, a previous study identified a coumarin compound meranzin in the ethanol extract of the edible red macroalga *Bangia fuscopurpurea*, but only a few studies have investigated the novel bioactivities of this compound and its hydrate. In this study, we examined the potential effects of meranzin and its hydrate on glioblastoma (GBM) using artificial intelligence‐based in silico tools and in vitro models. We used DIGEP‐Pred 2.0 to perform enrichment analysis of proteins affected by the two test compounds in GBM. ADMETlab 3.0 was applied to evaluate their medicinal chemistry properties and to predict absorption, distribution, metabolism, excretion, and toxicity (ADMET) profiles. We then selected the compound with higher pharmacological potential for in vitro studies in the human GBM cell line. In addition, DIGEP‐Pred 2.0 was used to predict its effects on cancer stemness markers. Enrichment analysis suggested that both meranzin and its hydrate may have therapeutic potential for GBM. Based on the in silico comparisons from ADMETlab 3.0, meranzin hydrate was considered to have higher pharmacological potential. Meranzin hydrate exhibited cytotoxicity in U87 cells, but not in GBM8401 cells. In the U87 model, meranzin hydrate demonstrated anti‐invasion activity (IC_50_ = 33.12 μM), although no inhibitory effect on colony formation was observed. Furthermore, meranzin hydrate suppressed the cancer stem cell property of sphere formation (IC_50_ = 21.43 μM). Moreover, in silico results showed that meranzin hydrate may downregulate the protein expression of two cancer stemness markers, ALDH1A1 and NANOG, which was further validated by in vitro Western blot analysis. This study provides in silico assessments of the pharmacological potential, together with in vitro evidence of the anti‐malignancy activity of the hydrate of meranzin in a human GBM cell line.

## Graphical abstract

In U87 cells, meranzin hydrate showed cytotoxicity (MIC = 50 μM) and anti‐invasion activity (IC_50_ = 33.12 μM) but had no inhibitory effect on colony formation. It also suppressed the cancer stem cell property of sphere formation (IC_50_ = 21.43 μM). In silico analysis indicated that meranzin hydrate may downregulate the protein expression of ALDH1A1 and NANOG, consistent with in vitro Western blot results.

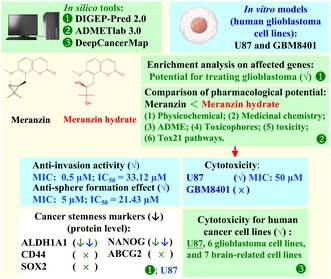

## 1 Introduction

### 1.1 Previous Research on the Bioactivities of *Bangia Fuscopurpurea*

The exploration of new bioactivities of marine‐derived compounds, including studies on their structural analogues, is an important research direction (Atanasov et al. 2021; Haque et al. 2022; Banerjee et al. 2022). Moreover, many research teams have focused on investigating natural products obtained from edible macroalgae (Cikoš et al. 2021; Arslan et al. 2025; Tufail et al. 2025), particularly red macroalgae (Aziz et al. 2020; Shih et al. 2017). *Bangia fuscopurpurea* is an edible red macroalga that is considered a common marine food in Asia. In 2021, it was recognized by the Ministry of Agriculture and Rural Affairs of China as one of “the ten excellent aquaculture germplasm resources” (Yan 2022). It has been cultivated in Putian (Fujian, China) for more than three decades (Huang 1991). Apart from its nutrient‐rich value, it has traditionally been regarded as potentially having functions in lowering blood pressure and reducing the risk of vascular diseases (Zheng et al. 2002). However, searches using the keyword “*Bangia fuscopurpurea*” (as of September 18, 2025) yielded only 29 articles in PubMed and 63 articles in Web of Science. This indicates that there is still clear potential for further exploration of its bioactivities. Previous studies on its bioactivities have mainly targeted two directions (Huang et al. 2024, 2020): (a) polysaccharides and (b) proteins, derived from its water extracts. Previous investigations of its bioactivities have largely focused on non‐cancer‐related aspects, and only a limited number of studies have explicitly addressed cancer‐related issues. Notably, two in vitro studies published in 2021–2022 reported the cytotoxicity of its polysaccharides in five ovarian cancer cell lines (Wu et al. 2021, 2020).

### 1.2 The Focus of This Study: A Coumarin Compound Meranzin Originating From *Bangia Fuscopurpurea* and Its Hydrate

Since 2020, four research teams have explored the bioactivities of the ethanol extracts from *B. fuscopurpurea* (Huang et al. 2020; Chang et al. 2023; Li et al. 2021) or its compound (phytol (Wang et al. 2023)). However, studies on the bioactivities of natural products from *B. fuscopurpurea* has mainly targeted the peripheral system, whereas studies directly addressing the nervous system, particularly central nervous system diseases, remain relatively limited (Huang et al. 2024). In addition, in 2024, we reported the in vitro neuroprotective effects of three phenolic acid derivatives originating from its ethanol extract in a Parkinson's disease model (Huang et al. 2024). In 2023, polyphenolic compounds in its ethanol extract, including a coumarin compound (meranzin), were structurally identified (Chang et al. 2023). In 1998, meranzin was reported to exhibit cytotoxicity against the human nasopharyngeal carcinoma cell line KB and the human bronchial epidermoid carcinoma cell line (NSCLC‐N6) (Kofinas et al. 1998). In 2006, meranzin hydrate was reported to exhibit antiproliferative activity against the mouse leukemia cell line L1210 and three human prostate cancer cell lines (LNCaP, PC3, and DU145) (Riviere et al. 2006). Although in vitro results from two previous studies suggest that meranzin and its hydrate may inhibit the viability of peripheral cancer cell lines, their effects on central nervous system‐related cancer cells, particularly brain cancer cells, remain unclear. Moreover, it is uncertain whether meranzin and its hydrate might influence other key characteristics of cancer cells, such as colony‐forming ability and invasive capacity. Therefore, we selected meranzin and its hydrate as the subjects of this study and investigated their potential effects on brain cancer.

### 1.3 Research Strategy for Investigating the In Vitro Anti‐Malignancy Activity of the Test Compound in Glioblastoma (GBM) Cells

In recent years, the possible anticancer activity of coumarins has attracted significant attention due to their promising bioactivity and potentially low toxicity (Küpeli Akkol et al. 2020), particularly in brain cancer (Shahbaz et al. 2025). However, the potential effects of coumarins on nervous system cancers are still not fully understood, particularly in brain cancer. Brain cancer is characterized by poor prognosis and high mortality rates in both pediatric and adult populations (Lin et al. 2020). Therefore, the continued development of new therapies for brain cancer remains urgently needed. Exploring the anti‐malignancy potential of compounds in brain cancer has become an important area of research in the field of food science and nutrition (Malik et al. 2025; Yang et al. 2025). Since cancer has been classified as a heterogeneous disease, recognizing its heterogeneity and diverse origins is crucial for developing optimal therapeutic strategies, particularly given the variations across tissue‐specific tumor types (Lenz et al. 2022). Tumor heterogeneity heightens the complexity and challenges of cancer therapy, rendering it almost impracticable to eradicate all cancer cell types with a single therapeutic method (Wang et al. 2019). For instance, propofol was reported to inhibit the malignancy of lung cancer cells but not that of brain cancer cells, and the design of in vitro experiments ruled out the possibility that the compound failed to efficiently cross the blood–brain barrier (BBB) (Hu et al. 2021). Moreover, brain cancer is considered a highly heterogeneous tumor (Lin et al. 2020). Therefore, it is difficult to infer from previous studies in other cancer cell line models whether meranzin and its hydrate possess cytotoxicity against brain cancer cells. Furthermore, inhibiting the characteristics of cancer stem cells (CSCs) remains a major challenge in cancer therapy. GBM is one of the most common primary brain cancers and harbors self‐renewing and tumorigenic CSCs, which contribute to tumorigenesis and treatment resistance (Lin et al. 2020; Lathia et al. 2015). Temozolomide, a chemotherapy drug, has been the standard first‐line treatment for GBM in the United States since 2005 (Tiek et al. 2022). However, an in vitro study using patient‐derived GBM biopsy samples in 2021 demonstrated that temozolomide could promote glioma stem cell formation (Gao et al. 2021). These findings indicate that clinical drugs with anticancer effects may still lead to the generation of cells exhibiting CSC characteristics. Therefore, in addition to traditional anti‐malignancy effects (cytotoxicity, inhibition of colony formation, and inhibition of invasion), the research strategy should also evaluate the effects of the test compound on the CSC properties of GBM cells. On the other hand, for drug development, it is critical to identify compounds with adequate bioactivity, suitable pharmacokinetic characteristics, and low toxicity (Sun et al. 2022; Dulsat et al. 2023). Consequently, during the early stages of drug development, in silico predictions of both medicinal properties and toxicity risks can increase the likelihood of successfully selecting compounds for further stages of development, such as lead optimization (Dulsat et al. 2023). It is still unclear whether meranzin or its hydrate may have higher pharmacological potential for GBM.

### 1.4 Experimental Design Using Artificial Intelligence (AI)–based In Silico Tools and In Vitro Models

The aim of this study is to evaluate the in silico pharmacological potential and the in vitro anti‐malignancy effect of the hydrate of the coumarin compound meranzin, originating from the edible macroalga (*B. fuscopurpurea*), in the human GBM cell line. In this study, we used the in silico tool DIGEP‐Pred 2.0 to explore the potential of meranzin and its hydrate in GBM. Using the in silico tool ADMETlab 3.0, we further predicted their medicinal chemistry properties and their absorption, distribution, metabolism, excretion, and toxicity (ADMET) profiles. Subsequently, we selected the compound with higher pharmacological potential for in vitro studies in the human GBM cell line, including cell viability assay, colony formation evaluation, cell invasion assay, and sphere formation test. Moreover, we used DIGEP‐Pred 2.0 to explore the potential effects of the test compound on the protein expression of cancer stemness markers in an in silico model, and we further validated these findings using in vitro Western blot analysis. Furthermore, with the in silico tool DeepCancerMap, we predicted the potential cytotoxic effects of the test compound in brain‐related human cancer cell lines.

## 2 Materials and Methods

### 2.1 Information on the Test Compounds

1. Meranzin (CAS: 23971–42‐8; SMILES: CC1 ([C@@H] (O1) CC2 = C (C=CC3 = C2OC (=O) C=C3) OC) C).
2. Meranzin hydrate (CAS: 5875‐49‐0; SMILES: CC (C) ([C@H] (CC1 = C (C=CC2 = C1OC (=O) C=C2) OC) O) O).

### 2.2 Potentially Relevant Literature on Meranzin and Its Hydrate Retrieved From Databases

This study searched for potentially relevant literature on meranzin and its hydrate in three databases (on September 18, 2025). (1) In PubMed, compound names were searched using the field “All Fields”. (2) For Web of Science, the same names were examined in the “Topic” field across all databases. (3) The PubChem Compound database was also consulted; compound names were retrieved and cross‐checked via “Consolidated References” under the “Literature” section.

### 2.3 In Silico Prediction of the Possible Effects of Meranzin and Its Hydrate on GBM

The in silico tool DIGEP‐Pred 2.0 (Ivanov et al. 2024) (Laboratory for Structure–Function Based Drug Design, Department of Bioinformatics, Institute of Biomedical Chemistry, Moscow, Russia) was used. The chemical structures of the two target molecules (meranzin and its hydrate) were input to predict their possible effects on gene expression profiles, using the dataset of protein expression changes from the Comparative Toxicogenomics Database (CTD). Structure–activity relationship (SAR) analysis and prediction for the test compounds was performed using the Prediction of Activity Spectra for Substances (PASS) technology. The “probability of being active (*Pa*)” represents the likelihood that a test compound belongs to the subcategory of active compounds, based on its structural similarity to representative molecules in the “active” subgroup of the PASS training set. The “probability of being inactive (*Pi*)” indicates the likelihood that a test compound is classified as an inactive compound, as determined by its structural resemblance to representative molecules in the “inactive” subgroup of the PASS training set. Only predicted activities with *Pa* > *Pi* were regarded as possible events. Enrichment analysis was conducted on genes affected by the two test compounds with *Pa* > *Pi* under either the downregulation or the upregulation model, using the DisGeNET database (Barcelona, Spain). This study then focused exclusively on GBM in DisGeNET. The odds ratio reflects the strength of the association between predicted genes and the disease. The *p*‐value indicates the statistical significance of this association. The adjusted *p*‐value (adj.*p*) was calculated from the *p*‐value using the Benjamini–Hochberg procedure for multiple testing correction.

### 2.4 In Silico Assessment of the Medicinal Chemistry Properties and ADMET of Meranzin and Its Hydrate

The “ADMET evaluation” function in the in silico tool ADMETlab 3.0 (Fu et al. 2024) (Xiangya School of Pharmaceutical Sciences, Central South University, Hunan, China) was used. The chemical structures of the two target molecules (meranzin and its hydrate) were input to assess their medicinal chemistry properties and ADMET. This study predicted (i) physicochemical properties (including radar charts), (ii) medicinal chemistry properties, (iii) absorption, (iv) distribution, (v) metabolism, (vi) excretion, (vii) toxicophore rules, (viii) toxicity, and (ix) Tox21 pathways. For the classification endpoints of predicted absorption, distribution, metabolism, and possible effects on Tox21 pathways, the prediction probability values were transformed into six symbols: 0–0.1 (−‐‐), 0.1–0.3 (−‐), 0.3–0.5 (−), 0.5–0.7 (+), 0.7–0.9 (++), and 0.9–1.0 (+++). These data were compared to summarize the advantages and disadvantages of the molecules for each item, which helped us select the compound with higher pharmacological potential for in vitro studies. In addition, four color labels were used to indicate performance: green = excellent, yellow = medium, red = poor, and gray = alerts. Differences in these color codes between the two compounds reflected their relative advantages and disadvantages for each item.

### 2.5 Cell Viability Assay

Vehicle (0.1% DMSO) or meranzin hydrate (catalog number TN1922, 99.49%, TargetMol) was prepared for in vitro experiments, which were performed in five independent replicates. Two human GBM cell lines, U87 (U‐87MG) and GBM8401 (GBM 8401), were used. Cells (3000 cells/well) were seeded into 96‐well plates and incubated overnight at 37°C with 95% air and 5% CO_2_. After treatment with the compound‐containing culture medium (RPMI1640 supplemented with 10% FBS) for 24 h, Alamar Blue reagent was added at a ratio of culture medium to reagent of 10:1 (Invitrogen) (Chu et al. 2022), and the cells were incubated at 37°C for 3 h. Absorbance was measured at 570 and 620 nm using a microplate reader (Dynex Technologies Inc). Cell viability was calculated by subtracting the absorbance at 620 nm from that at 570 nm for each sample, according to the following formula:

$$\text{Cell viability}\;\left(\%\right)=\left(\text{Experimental group}/\text{Control}\;\left(\text{vehicle}\right)\;\text{group}\right)\times 100\%.$$

### 2.6 Colony Formation Evaluation

A previously published method was modified to perform the colony formation evaluation (Chu et al. 2022; Wang et al. 2022). Cells (3000 cells/well) were seeded into 6‐well plates and treated with vehicle (0.1% DMSO) or meranzin hydrate (0.5–50 μM) for 10 days in three independent experiments. After treatment, the culture medium was removed, and the cells were washed with PBS. Subsequently, the cells were fixed with 4% paraformaldehyde (Sigma‐Aldrich) in PBS for 30 min. After removing the fixative, the cells were stained with 0.5% crystal violet solution (Sigma‐Aldrich) for 2 h. Excess crystal violet was carefully removed with a pipette, and the wells were immersed in distilled water for 1 min to wash off the residual dye. Finally, the 6‐well plates were scanned with a scanner, and the number of colonies containing more than 50 cells was counted using ImageJ software.

$$\text{Colony formation}\;\left(\%\right)=\left(\text{Experimental group}/\text{Control}\;\left(\text{vehicle}\right)\;\text{group}\right)\times 100\%.$$

### 2.7 Cell Invasion Assay

A previously published method was modified to conduct the cell invasion assay (Chu et al. 2022). Cells (1 × 10^5^ cells, 50 μL/well) were seeded into the upper chamber of a Transwell invasion assay device with serum‐free medium containing vehicle (0.1% DMSO) or meranzin hydrate (0.5–50 μM) in three independent experiments. The lower chamber was supplemented with 30 μL of medium containing 10% FBS. A polycarbonate membrane with 8‐μm pores (Nucleopore; Costar), coated with diluted Matrigel (BD Biosciences; Matrigel: serum‐free medium = 1:3), was used to allow cell adhesion, and this coated membrane separated the upper and lower chambers. After incubation at 37°C in 5% CO_2_ for 24 h, the cells that had migrated to the lower surface of the membrane were fixed with anhydrous methanol for 1 h and stained with 10% Giemsa solution (Sigma‐Aldrich) for 1 h. A microscopic imaging system (Leica) was used to photograph and count the cells in each well, with five random areas per well quantified using ImageJ software (National Institutes of Health, Bethesda, MD, USA).

$$\text{Cell invasion}\;\left(\%\right)=\left(\text{Experimental group}/\text{Control}\;\left(\text{vehicle}\right)\;\text{group}\right)\times 100\%.$$

### 2.8 Sphere Formation Test

A previously reported method was modified to carry out the sphere formation test (Chu et al. 2022). Cells were suspended in serum‐free DMEM/F12 medium (Gibco) supplemented with B‐27 (Gibco), EGF (20 ng/mL; PeproTech), and bFGF (20 ng/mL; PeproTech). A total of 1000 cells/well were seeded into ultralow attachment 24‐well plates (Corning Life Sciences; total volume = 0.5 mL) and treated with vehicle (0.1% DMSO) or meranzin hydrate (0.5–50 μM) in three independent experiments. GBM cells were maintained under humidified conditions at 37°C in 5% CO_2_ and 95% air to promote tumor sphere formation. After 7 days, spheres were observed and counted using an optical microscope (20× objective; Leica).

$$\text{Sphere formation}\;\left(\%\right)=\left(\text{Experimental group}/\text{Control}\;\left(\text{vehicle}\right)\;\text{group}\right)\times 100\%.$$

### 2.9 In Silico Predictions of the Possible Effects of Meranzin and Its Hydrate on Cancer Stemness Markers

Using the in silico tool DIGEP‐Pred 2.0 (Ivanov et al. 2024) with a cut‐off of *Pa* set to “no limit”, we predicted the possible effects of meranzin and its hydrate on gene expression profiles with the dataset of protein expression changes from CTD under the downregulation or upregulation model. This study focused exclusively on cancer stemness markers from CTD at the protein level. The invariant Accuracy of Prediction (IAP) represents the average prediction accuracy for the entire PASS training set through leave‐one‐out cross‐validation. Based on previous studies related to cancer stemness markers (Ji et al. 2021; Kim et al. 2021; Le et al. 2020; Wu et al. 2022), we selected five protein targets in the downregulation model (cluster of differentiation 44 (CD44), aldehyde dehydrogenase 1 family member A1 (ALDH1A1), nanog homeobox (NANOG), ATP‐binding cassette sub‐family G member 2 (ABCG2), and SRY‐box transcription factor 2 (SOX2)) and four protein targets in the upregulation model (CD44, ALDH1A1, NANOG, and ABCG2). The possible effects of meranzin and its hydrate on each cancer stemness marker were assessed by DIGEP‐Pred 2.0, which provided probability values for each event in terms of *Pa* and *Pi*. Only predicted activities with *Pa* > *Pi* were regarded as possible events.

### 2.10 Western Blot Analysis

Western blot analysis was performed with modifications from previously reported methods (Chu et al. 2022; Wang et al. 2022). The total protein lysate of GBM cells was extracted using PRO‐PREP Protein Extraction Solution (Catalog no. 17081.1; iNtRON Biotechnology, Seongnam, Korea) for 30 min at 4°C. After centrifugation at 14,000 rpm for 30 min at 4°C (Microfuge 22R Centrifuge; Beckman Coulter, Fullerton, CA), the supernatant was collected. Protein concentrations were determined using a bicinchoninic acid (BCA) protein assay kit (Catalog no. 23227; Pierce Biotechnology, Rockford, IL). Sample buffer (0.3 M Tris–HCl, pH 6.8; 50% glycerol; 10% sodium dodecyl sulfate (SDS); 0.5% NaN_3_; 0.05% bromophenol blue; and 5% 2‐mercaptoethanol) was added to each sample at a ratio of 4:1 and boiled for 5 min at 95°C. Equal amounts of protein (20 μg) were separated by SDS–polyacrylamide gel electrophoresis (SDS‐PAGE, 8%–12%) at 80 V for 2.5 h and transferred onto polyvinylidene fluoride (PVDF) membranes (Catalog no. IEVH85R; EMD Millipore, Billerica, MA) at 200 mA for 2 h at 4°C. Non‐specific binding was blocked with 5% milk in Tris‐buffered saline containing Tween 20 (TBST) for 30 min at room temperature. The PVDF membranes were incubated overnight at 4°C with the following primary antibodies: ALDH1A1 (Catalog no. GTX123973; 1:3000; GeneTex), NANOG (Catalog no. GTX100863; 1:1000; GeneTex), and β‐actin (Catalog no. sc‐47,778; 1:500; Santa Cruz Biotechnology). After washing, the membranes were incubated for 90 min at room temperature with horseradish peroxidase (HRP)‐conjugated secondary antibodies (1:5000 dilution in 5% milk in TBST; EMD Millipore). The secondary antibodies used were goat anti‐mouse IgG (Catalog no. AP124P; EMD Millipore), and goat anti‐rabbit IgG (Catalog no. AP132P; EMD Millipore). The luminescent signals of protein targets on the PVDF membranes were detected using the SuperSignal West Pico PLUS Chemiluminescent Substrate (Catalog no. 34580; Thermo Fisher Scientific, Waltham, MA) and visualized with the KETA gel documentation system (Neo‐One Bio Life Science, Taipei, Taiwan). The signal intensities were quantified using ImageJ software.

### 2.11 In Silico Prediction of the Possible Cytotoxic Effects of Meranzin and Its Hydrate on Brain‐Related Human Cancer Cell Lines

We used the “activity predicting for cancer cell lines” function within the “activity predicting” module of the in silico tool DeepCancerMap (Wu et al. 2023) (idrugLab, South China University of Technology, Guangzhou, China). The chemical structures of the two target molecules (meranzin and its hydrate) were input to predict their possible cytotoxic effects. We further analyzed the prediction results (predicted scores) for brain‐related human cancer cell lines and recorded the area under the receiver operating characteristic (ROC) curve (AUC) for each model along with its training data size. DeepCancerMap originally provided 25 brain‐related human cancer cell line models. Two databases, the American Type Culture Collection (ATCC; Virginia, USA) and Cellosaurus (Bairoch 2018) (Swiss Institute of Bioinformatics, University of Geneva, Switzerland), were used to verify and correct the species origin, tissue origin, and disease type of each cell line in the prediction results. After our confirmation, DeepCancerMap included 23 brain‐related human cancer cell line models. In addition, we also presented the predicted scores of the compounds in two additional non‐brain‐related human cancer cell line models. Based on our in vitro results in U87 cells, we set a threshold for the predicted in silico scores in other human cancer cell lines from DeepCancerMap.

### 2.12 Statistical Analysis and Other Methodologies

All in vitro experimental results are presented as mean ± standard deviation (SD). Intergroup comparisons were performed using the unpaired *t*‐test. *p < 0.05 and **p < 0.01 were considered statistically significant. By the definition of the minimum inhibitory concentration (MIC; the lowest concentration that produces a specific bioactivity) (Van Dijck et al. 2018), we assessed the potential in vitro anti‐malignancy activity of the test compound. An interpolation method was used to determine the half maximal inhibitory concentration (IC_50_) for in vitro studies. The structural editor InDraw (Integle, Shanghai, China) was used to illustrate the chemical structures of meranzin and its hydrate. The graphical abstract was designed using the Figdraw platform (ID: UISAAccf4a; Figdraw 2.0; Home for Researchers, Zhejiang, China).

## 3 Results

### 3.1 Potential of Meranzin and Its Hydrate for Treating GBM by Enrichment Analysis of Proteins

To the best of our knowledge, based on a literature search, no studies have explored the potential effects of meranzin and its hydrate on GBM. Using the in silico tool DIGEP‐Pred 2.0, we performed enrichment analysis of compound‐affected genes at the protein level (with *Pa* > *Pi*) under the downregulation or upregulation model for GBM, using data from the DisGeNET database. In enrichment analysis, a disease with adj.*p* < 0.05 and associated with at least two predicted genes for the test compound was considered significantly enriched (Ivanov et al. 2024). Predictions from both the downregulation and upregulation models suggested that meranzin and its hydrate may have potential for treating GBM, GBM multiforme, and giant cell GBM (Table S1).

### 3.2 Predictions of the Medicinal Chemistry Properties and ADMET of Meranzin and Its Hydrate

We used the in silico tool ADMETlab 3.0 to generate radar charts of the physicochemical properties of meranzin and its hydrate (Figure S1), and their detailed data are shown in Table S2. Moreover, we compared their predicted medicinal chemistry properties (Table S3). We further presented predictions for their absorption and distribution (Table S4), metabolism, and excretion (Table S5). We also reported results from toxicophore rule analysis (Table S6), toxicity predictions (Table S7), and possible effects on the Tox21 pathways (Table S8). Based on in silico comparisons from ADMETlab 3.0, meranzin hydrate might have higher pharmacological potential. Therefore, we selected meranzin hydrate for in vitro studies in the human GBM cell line.

### 3.3 In Vitro Effects of Meranzin Hydrate on the Characteristics of Human GBM Cells

**FIGURE 1:**
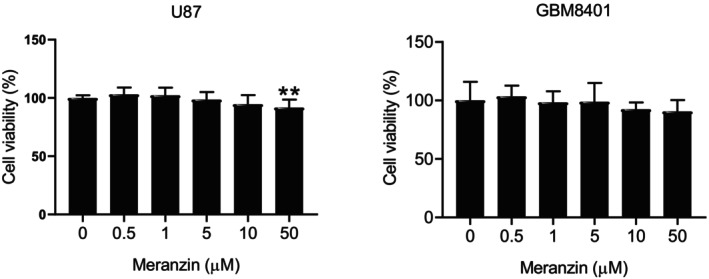
Effect of meranzin hydrate (Meranzin) on the viability of U87 and GBM8401 cells. After 24 h treatment, meranzin hydrate significantly reduced the viability of U87 cells at 50 μM, but had no significant effect on the viability of GBM8401 cells at concentrations of 0.5–50 μM (*n* = 5). In the U87 model, meranzin hydrate exhibited cytotoxicity. **p < 0.01 vs. control (vehicle) group.

**FIGURE 2:**
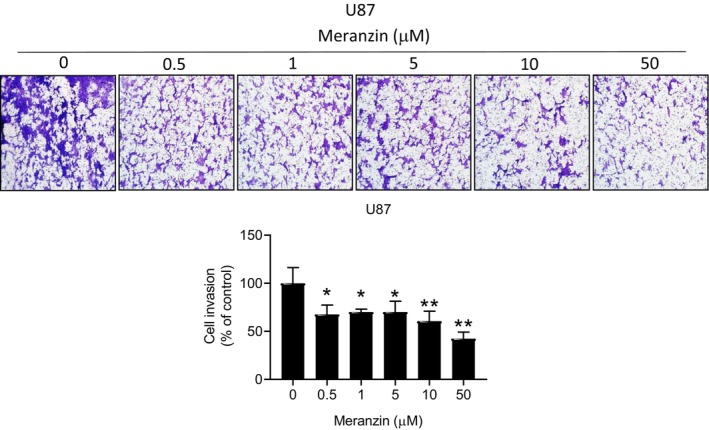
Effect of meranzin hydrate (Meranzin) on the invasion activity of U87 cells. After 24 h treatment, meranzin hydrate significantly inhibited the invasion activity (0.5–50 μM). (*n* = 3) In the U87 model, meranzin hydrate exerted anti‐invasion activity (IC_50_ = 33.12 μM). *p < 0.05, **p < 0.01 vs. control (vehicle) group.

**FIGURE 3:**
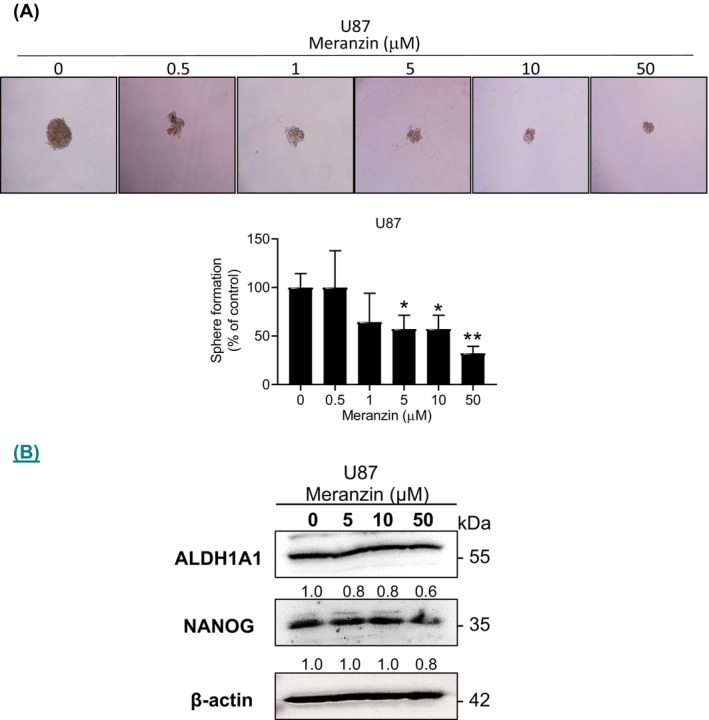
Effect of meranzin hydrate (Meranzin) on the cancer stemness property of U87 cells. (A) After 7 days of treatment, meranzin hydrate significantly reduced sphere formation (5–50 μM; IC_50_ = 21.43 μM). (*n* = 3) *p < 0.05, **p < 0.01 vs. control (vehicle) group. (B) Western blot analysis of two cancer stemness markers. After 24 h of treatment, meranzin hydrate clearly reduced the protein expression of ALDH1A1 and NANOG.

In U87 cells, meranzin hydrate significantly decreased cell viability at a MIC of 50 μM after 24 h of treatment compared with the control (vehicle) group. However, meranzin hydrate had no effect on the viability of GBM8401 cells after 24 h of treatment (Figure 1). We further explored the potential effects of the compound on other characteristics of human GBM cells. Compared with the control group, meranzin hydrate did not affect colony formation in GBM8401 cells after 7 days of treatment (Figure S2). Meranzin hydrate significantly inhibited invasion activity in U87 cells at a MIC of 0.5 μM after 24 h of treatment compared with the control group (Figure 2). Meranzin hydrate also significantly reduced sphere formation in U87 cells at a MIC of 5 μM after 7 days of treatment compared with the control group (Figure 3A).

### 3.4 Predicted and Validated Effects of Meranzin Hydrate on Cancer Stemness Markers

We used the in silico tool DIGEP‐Pred 2.0 to predict the effects of meranzin hydrate on cancer stemness markers (Table 1). Only activities with *Pa* > *Pi* were regarded as possible events (Ivanov et al. 2024). We set the threshold for *Pa* at 0.500 for both the downregulation and upregulation models. Meranzin hydrate might downregulate the protein expression of ALDH1A1 and NANOG (but not SOX2) and might not affect CD44 or ABCG2. In contrast, meranzin might downregulate only the protein expression of NANOG. We next performed Western blot analysis to examine the effects of meranzin hydrate on two cancer stemness markers (ALDH1A1 and NANOG) in GBM cells (Figure 3B). After 24 h of treatment, meranzin hydrate reduced the protein expression of ALDH1A1 (MIC = 5 μM) and NANOG (MIC = 50 μM) in U87 cells. In addition, meranzin hydrate (5–50 μM) did not markedly affect the protein expression of β‐actin in U87 cells.

**TABLE 1:** Predicted effects of meranzin and its hydrate on cancer stemness markers at the protein level.

I. Downregulation model								
No.	Genes	IAP	Meranzin			Meranzin hydrate		
			Pa	*Pi*	*Pa* > *Pi*, *Pa* ≧ 0.5	Pa	*Pi*	*Pa* > *Pi*, *Pa* ≧ 0.5
1	CD44	0.873	0.477	0.011	No	0.498	0.009	No
2	ALDH1A1	0.875	0.436	0.072	No	0.642	0.023	Yes
3	NANOG	0.770	0.548	0.020	Yes	0.506	0.029	Yes
4	ABCG2	0.876	0.260	0.123	No	0.428	0.050	No
5	SOX2	0.900	0.320	0.030	No	0.282	0.037	No

II. Upregulation model								
No.	Genes	IAP	Meranzin			Meranzin hydrate		
			*Pa*	*Pi*	*Pa* > *Pi*, *Pa* ≧ 0.5	*Pa*	Pi	*Pa* > *Pi*, *Pa* ≧ 0.5
1	CD44	0.853	0.346	0.101	No	0.268	0.193	No
2	ALDH1A1	0.794	0.426	0.087	No	0.416	0.094	No
3	NANOG	0.959	0.169	0.101	No	0.161	0.109	No
4	ABCG2	0.829	0.368	0.076	No	0.435	0.039	No

*Note:* Shaded cells indicate positive predictions that meet the criteria (Pa > Pi and Pa ≥ 0.5). Boldface highlights key results that meet the predefined selection criteria.

### 3.5 Predicted Cytotoxic Effects of Meranzin Hydrate on Brain‐Related Human Cancer Cell Lines

Using the in silico tool DeepCancerMap, we predicted possible cytotoxic effects of meranzin hydrate on brain‐related human cancer cell lines (Table 2). We set the threshold for the predicted score at 0.500. Meranzin hydrate might exert cytotoxic activity against U87 and 13 other brain‐related human cancer cell lines, whereas meranzin might show cytotoxic activity against U87 and 12 other brain‐related human cancer cell lines.

**TABLE 2:** Predicted cytotoxic effects of meranzin and its hydrate in brain‐related human cancer cell lines.

	Tissue	Disease	Model	Model AUC	Training data size	Predicted score	
						Meranzin	Meranzin hydrate
1^B^	Brain	GBM	A 172	0.8462	208	**0.977**	**0.974**
2^B^			U‐87 MG	0.867	755	**0.964**	**0.835**
3^B^			U87	0.6146	200	**0.677**	**0.674**
4^B^			SNB‐75	0.7684	1050	**0.952**	**0.866**
5^B^			SF‐126	0.7917	136	**0.875**	**0.886**
6^B^			XF498	0.8632	238	**0.866**	**0.737**
7^A^			LN‐229	1	30	0.482	0.454
8^B^			T98G	0.9443	549	0.304	**0.763**
9^B^			SNB‐78	1	83	0.148	0.164
10^A^			DBTRG‐05MG	0.6667	106	0.114	0.123
11^B^			SF‐295	0.8541	1288	0.058	**0.855**
12^B^		Gliosarcoma	SF‐539	0.7635	1367	**0.913**	**0.641**
13^A^		Glioma; oligodendroglioma	Hs 683	0.7222	169	0.002	0.002
14^B^		Astrocytoma	U373 MG	0.9844	247	**0.942**	**0.709**
15^B^			SNB‐19	0.8926	899	**0.869**	**0.902**
16^A^			SW1088	0.8	111	**0.858**	**0.816**
17^B^			U‐251	0.864	1970	**0.829**	0.175
18^B^			SF‐268	0.8739	2019	0.229	0.133
19^A^		Neuroglioma	H4	0.9722	134	**0.919**	**0.925**
20^A^		Neuroblastoma	SK‐N‐SH	0.9362	553	0.235	0.116
21^A^			IMR‐32	0.9901	349	0.029	0.019
22^A^		Neuroepithelioma	SK‐N‐MC	1	155	**0.824**	**0.845**
23^A^	Brain; cerebellum	Desmoplastic cerebellar medulloblastoma	Daoy	0.7143	154	**0.559**	**0.702**
24^A^	Bone	Osteosarcoma	SJSA‐1	0.8889	507	**0.984**	**0.979**
25^B^	Pleural effusion	Plasma cell myeloma; multiple myeloma	NCI‐H929	1	37	0	0

*Note:* Gray shading indicates predicted cytotoxicity with a score ≥ 0.500. This cutoff was used to identify potential cytotoxic effects in cancer cell lines. Boldface highlights key results that meet this cutoff.

Abbreviations: A, ATCC; B, Cellosaurus.

## 4 Discussion

### 4.1 Potential for Treating GBM and In Silico Predictions of the Pharmacological Potential of Meranzin and Its Hydrate

The limited number of publications on meranzin (originating from *B. fuscopurpurea* (Chang et al. 2023)) and its hydrate indicates that there remains considerable scope for further research on these two compounds in the field of cancer research (Table 3). During the early phase of drug discovery, searching for active compounds for a target can still be both time‐ and resource‐intensive, whereas in silico prediction of bioactivity can refine the compound screening process in a more efficient and refined manner (Fredin Haslum et al. 2024). Predictions at the protein level from DIGEP‐Pred 2.0 suggested that meranzin and its hydrate may have potential for treating GBM, GBM multiforme, and giant cell GBM (Table S1). By reviewing clinical trial data, Sun et al. (Sun et al. 2022) concluded that four possible causes accounted for 90% of clinical failures of drug development: (i) poor clinical therapeutic effects (40%–50%), (ii) toxicities (unmanageable or unexpected; 30%), (iii) poor drug‐like properties (10%–15%), and (iv) lack of commercial needs or weak strategic planning (10%). We used ADMETlab 3.0 to perform in silico predictions of the pharmacological potential of meranzin and its hydrate. Many indicators are used to evaluate the pharmacological potential of compounds. Here are some examples. The BBB can limit the entry of compounds from the peripheral system and block their possible effects on the central nervous system (Cheng et al. 2022). Cytochrome P450 (CYP) enzymes play an important role in the pharmacokinetics of compounds. CYP enzymes are responsible for the metabolism of over 75% of marketed drugs (Gonzalez et al. 2021), and five human CYP isoforms (1A2, 2C19, 2C9, 2D6, and 3A4) contribute to ~95% of CYP‐mediated drug metabolism (Goldwaser et al. 2022). To compare the predicted pharmacological potential of meranzin and its hydrate, we summarized the advantages and disadvantages of meranzin hydrate relative to meranzin in Table 3. Therefore, meranzin hydrate was selected for in vitro studies.

**TABLE 3:** Summary of experimental results for meranzin and its hydrate.

	Compounds		Meranzin	Meranzin hydrate
Previous studies	Chemical structures		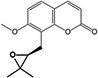	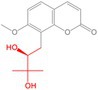
	Current status		Originating from the edible *Bangia fuscopurpurea*	The hydrate of meranzin
	The number of potential references	**WOS; PubMed**	197; 63	170; 49
		**PubChem**	10	14
	Bioactivities associated with cancer		Cytotoxicity in the nasopharyngeal carcinoma and bronchial epidermoid carcinoma cell lines (Kofinas et al. 1998).	Antiproliferative activity in a leukemia cell line and three prostate cancer cell lines (Riviere et al. 2006).
This study	(1) Therapeutic potential for GBM	In silico model	Enrichment analysis of proteins: Yes	Enrichment analysis of proteins: Yes
	(2) Physicochemical properties	**Advantages of both compounds**	The predicted values were within the optimal range.	
	(3) Predicted medicinal chemistry properties	**Advantages of meranzin hydrate**	Better QED; lower probability of being a promiscuous compound.	
		Disadvantages of meranzin hydrate	Lower MCE‐18 value.	

	(4) Predictions of absorption, distribution, metabolism, and excretion	**Advantages of meranzin hydrate**	Predicted higher intestinal membrane permeability; higher probabilities of achieving ≥ 20%, ≥ 30%, or ≥ 50% bioavailability (BAB); lower probabilities of acting as inhibitors (CYP1A2, CYP2C8) or substrates (CYP1A2, CYP2C19, CYP2C9, CYP3A4, CYP2B6).	
		Disadvantages of meranzin hydrate	Higher probability of being an inhibitor of CYP2C19; higher probability of being a substrate of CYP2D6.	
	(5) Predictions based on toxicophore rules	**Advantages of meranzin hydrate**	Fewer alerts for the genotoxic carcinogenicity mutagenicity rule, nongenotoxic carcinogenicity rule, skin sensitization rule, nonbiodegradable rule, SureChEMBL rule, and FAF‐Drugs4 rule.	
	(6) Predicted toxicities	**Advantages of meranzin hydrate**	Lower probabilities of toxicity for drug‐induced liver injury, Ames toxicity, skin sensitization, carcinogenicity, eye corrosion, drug‐induced nephrotoxicity, and hematotoxicity.	
		Disadvantages of meranzin hydrate	Higher probability of being a respiratory toxicant and of causing ototoxicity.	
	(7) Predicted effects on Tox21 pathways	**Advantages of meranzin hydrate**	Lower probabilities of being active in the androgen receptor, aromatase, antioxidant response element, ATPase family AAA domain‐containing protein 5, heat shock factor response element, mitochondrial membrane potential, and p53 pathways.	
	(8) Cytotoxicity in human brain cancer cell lines	U87	—	**MIC = 50 μM**
		GBM8401	—	No
		In silico model	U87 and 12 other cell lines.	U87 and 13 other cell lines.
	(9) Anti‐colony formation	GBM8401	—	No
	(10) Inhibition of invasion	U87	—	**MIC = 0.5 μM; IC**_**50**_ **= 33.12 μM**
(11) Sphere formation inhibition	U87	—	**MIC = 5 μM; IC**_**50**_ **= 21.43 μM**

	(12) Downregulation of cancer stemness markers (protein level)	In silico model	**(↓): NANOG**; (×): ALDH1A1, CD44, ABCG2, SOX2.	**(↓): ALDH1A1, NANOG**; (×): CD44, ABCG2, SOX2.
U87		—	**(↓): ALDH1A1 (MIC = 5 μM)** **(↓): NANOG (MIC = 50 μM)**

*Note:* —: no experiment.

### 4.2 The New Bioactivity of the Hydrate of Meranzin: In Vitro Anti‐Malignancy Activity in the GBM Cell Line

In the U87 model, meranzin hydrate exhibited cytotoxicity (Figure 1) and anti‐invasion activity (Figure 2) and inhibited the CSC property (sphere formation; Figure 3A). Our in vitro data above may be consistent with predictions from DIGEP‐Pred 2.0. We further explored the possible effects of meranzin hydrate on GBM at the protein level. In the in silico model, meranzin hydrate might downregulate the protein expression of ALDH1A1 and NANOG, whereas meranzin might downregulate only NANOG (Table 1). These in silico predictions could provide useful hints for future in vitro experiments, indicating which protein targets should be prioritized for testing the possible effects of meranzin and its hydrate on cancer stemness markers. Consistent with the in silico predictions, our Western blot analysis demonstrated that meranzin hydrate reduced the protein expression of ALDH1A1 and NANOG in U87 cells after 24 h of treatment (Figure 3B). In addition, we found that the datasets of DIGEP‐Pred 2.0 still lacked data on SOX2 (upregulation model), prominin‐1 (CD133), octamer‐binding transcription factor 4 (OCT4), and nestin. Since these four proteins are also cancer stemness markers (Ji et al. 2021; Kim et al. 2021; Le et al. 2020; Wu et al. 2022), it is recommended that the research group of DIGEP‐Pred 2.0 consider incorporating these targets into their datasets in future studies.

### 4.3 Comparative Evaluation of the In Vitro Anti‐Malignancy Activity of the Hydrate of Meranzin in GBM Cell Lines

Studying the SARs of coumarins has been considered a key step for further optimizing their bioactivities (Kubrak et al. 2025). Compared with other types of cancer cells, the number of studies investigating the application of coumarin compounds in brain cancer remains relatively limited. A review published in 2024 reported only five coumarins that had been studied in GBM (Lianou et al. 2024). For instance, a 2024 meta‐analysis of 27 in vitro anticancer studies on two coumarins (auraptene and umbelliprenin) identified only three GBM‐related reports (Shakiba and Rassouli 2024). Although research on the application of coumarin‐based structural analogues in GBM is still limited, our literature search identified three studies that examined the possible cytotoxicity of other coumarin‐based analogues in GBM cell lines. A natural coumarin, osthole, isolated from Umbelliferae plant monomers (ripe fruit), shares the same core structure as meranzin hydrate. Osthole at 50 μM reportedly had no significant effect on U87 cell viability at 24 h but significantly reduced it at 72 h (Lin et al. 2015). In contrast, meranzin hydrate at 50 μM significantly reduced U87 cell viability at 24 h (Figure 1), representing an advantage of meranzin hydrate over its structural analogue osthole. In addition, osthole (10–100 μM) was found to have no significant cytotoxicity in GBM8401 cells at 24 h (Lin et al. 2014), and similarly, meranzin hydrate (0.5–50 μM) showed no significant effect on GBM8401 cell viability at 24 h (Figure 1). In 2025, the MIC of the cytotoxic effect of another coumarin‐based analogue, umbelliferone, on U87 cells at 24 h was reported to be 5 μM (Ma et al. 2025), while that of meranzin hydrate was 50 μM (Figure 1). Comparative evaluation of the in vitro cytotoxicity of meranzin hydrate in GBM cells against other coumarin compounds could provide valuable insights for future SAR studies. To the best of our knowledge, however, there remains a lack of experimental data on other coumarin‐based structural analogues in U87 and GBM8401 cells, including assays for colony formation, cell invasion, and sphere formation. Therefore, it is recommended that future studies further investigate these aspects to enhance understanding of the SARs of coumarin‐based analogues and to provide guidance for their structural optimization.

### 4.4 Predicted Effects of the Hydrate of Meranzin on Brain‐Related Human Cancer Cell Lines

**FIGURE 4:**
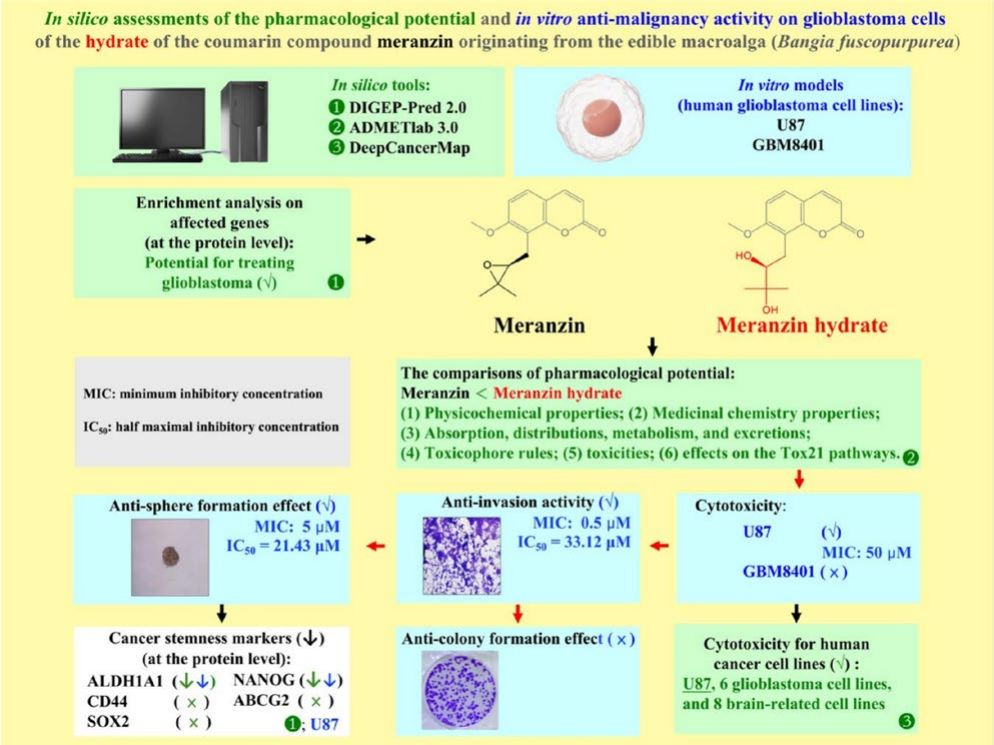
Summary of the findings of this study. This figure summarizes the in silico assessments of pharmacological potential and the in vitro anti‐malignancy activity in the human GBM cell line of the hydrate of the coumarin compound meranzin originating from the edible macroalga *Bangia fuscopurpurea*.

Cancer cell lines have long served as an important experimental framework for the exploration of new anticancer drugs (Ruiz‐Moreno et al. 2018; Mirabelli et al. 2019). Although all cancer cell lines share some common characteristics, each still has distinct genotypic and phenotypic properties, which may result in different responses to a drug (Ruiz‐Moreno et al. 2018). According to the ATCC database, at least 83 brain‐related human cancer cell lines have been recorded, including GBM (62), oligodendroglioma (2), astrocytoma (4), neuroblastoma (9), and medulloblastoma (6). Therefore, screening all of these cell lines for the potential cytotoxic effects of meranzin hydrate would be highly time‐ and cost‐intensive. Some research groups have utilized such tools to predict the potential cytotoxic effects of compounds, which could provide in silico guidance for refining the experimental designs of future in vitro studies (Cheng et al. 2024). The datasets of DeepCancerMap contain data from 23 types of brain‐related human cancer cell lines (Wu et al. 2023). We used DeepCancerMap and found that meranzin hydrate might exhibit cytotoxic activity against 6 GBM cell lines, 1 gliosarcoma cell line, 3 astrocytoma cell lines, 1 neuroglioma cell line, 1 neuroepithelioma cell line, and 1 desmoplastic cerebellar medulloblastoma cell line (Table 2). These in silico predictions could provide the hints for future in vitro experiments, indicating which cell lines should be prioritized for testing the potential cytotoxic effects of meranzin hydrate in other brain‐related human cancer cell lines. In addition, using the ATCC and Cellosaurus databases, we verified the tissue origin of each cell line from the brain‐related prediction results of DeepCancerMap and revised the tissue origin annotations of SJSA‐1 and NCI‐H929. Our in vitro data supported the notion that DeepCancerMap could be useful for in silico screening of compounds with cytotoxicity against the GBM cell line. We recommend that the research team of DeepCancerMap examine and correct these issues in the future. Additionally, we found that meranzin and its hydrate were predicted to exert cytotoxic activity in an osteosarcoma cell line (SJSA‐1). Furthermore, we summarized our experimental data from in silico and in vitro models in Figure 4 and Table 3.

### 4.5 Recommendations for Future Research Directions

The discovery of novel anticancer drugs continues to draw significant attention and remains a challenging endeavor (Wu et al. 2023), especially for GBM. For drug development, compounds from natural products still require both continuous and cost‐feasible production to obtain sufficient quantities (Atanasov et al. 2021), particularly marine‐derived compounds (Huang et al. 2012). Meranzin has been identified in *B. fuscopurpurea* (Chang et al. 2023), which could serve as a stable cultured source for compound supply. In addition, the synthetic accessibility score and GASA of meranzin and its hydrate indicated easy accessibility (Table S3). Moreover, there are already commercial companies that offer meranzin and its hydrate with ≥ 95% purity. The limitations of this study may lie in the fact that only in vitro models and in silico tools were used to investigate the anti‐malignancy activity of the hydrate of meranzin against GBM and to evaluate its predicted pharmacological potential, without data from in vivo models. In addition, it should be noted that four parameters of the hydrate of meranzin were not within the appropriate range for the predicted medicinal chemistry properties (Table S3). Some researchers have argued that multiple definitions of medicinal chemistry properties (such as drug‐likeness) might substantially limit the potential for molecular diversity in drug discovery (Rishton 2008). This study suggested four possible issues of the hydrate of meranzin for future research. (i) Only a few studies have explored the possible effects of coumarins on the CSC properties of GBM; therefore, it would be worthwhile to investigate the effects of meranzin hydrate on other cancer stemness markers and molecular pathways in in vitro models of GBM. (ii) To examine the anti‐malignancy activity of meranzin hydrate in in vivo models of GBM, especially regarding the inhibition of CSC phenotypes. (iii) Synergistic effects of natural compounds and clinical chemotherapeutic agents have been recognized as important strategies in cancer treatment (Castañeda et al. 2022). Therefore, it may be worthwhile to explore the potential effects of meranzin hydrate in combination with chemotherapeutic agents against GBM. (iv) To explore the possible effects of meranzin hydrate in other brain‐related human cancer cell lines beyond GBM. (v) To investigate ethnic differences in the susceptibility of GBM cells to the effects of meranzin hydrate. According to the Bioresource Collection and Research Center (BCRC; Hsinchu, Taiwan) database, U87 (BCRC no. 60360) originated from a Caucasian individual, whereas GBM8401 (BCRC no. 60163) originated from an Asian (Chinese) individual. We demonstrated that meranzin hydrate exhibited cytotoxicity in U87 cells but not in GBM8401 cells. Additionally, we found that the datasets of DeepCancerMap still lacked data on GBM8401. It is recommended that the research group of DeepCancerMap consider incorporating GBM8401 into their datasets in future studies. (vi) It would also be of interest to examine the possible effects of meranzin hydrate on non‐GBM cancer cell lines, such as the osteosarcoma cell line SJSA‐1. We expect that this study will promote research on the bioactivities of the hydrate of meranzin originating from the edible macroalga *B. fuscopurpurea* for GBM and provide knowledge from the perspective of molecular nutrition to better understand its possible effects on GBM cells.

## 5 Conclusions

Based on in silico comparisons (ADMETlab 3.0, DIGEP‐Pred 2.0, and DeepCancerMap), the results suggest that meranzin hydrate may have higher pharmacological potential for GBM than meranzin (originating from *B. fuscopurpurea*). This study also demonstrated the in vitro anti‐malignancy activity of the hydrate of meranzin in the GBM cell line. Furthermore, meranzin hydrate inhibited the CSC property of U87 cells (sphere formation) with a MIC of 5 μM (IC_50_ = 21.43 μM). We expect that this study will promote further research on the bioactivities and cellular mechanisms of meranzin hydrate in brain cancers, particularly GBM, and inspire scientists to explore the potential of other coumarin compounds from the edible macroalga *B. fuscopurpurea*.

## Author Contributions

**Shi‐Ying Huang:** conceptualization (equal), formal analysis (equal), investigation (equal), methodology (equal), writing – original draft (equal), writing – review and editing (equal). **Yi‐Chen Chang:** conceptualization (equal), formal analysis (equal), investigation (equal), methodology (equal), writing – original draft (equal), writing – review and editing (equal). **Charles Chien‐Chih Chiu:** investigation (supporting), writing – original draft (supporting). **Chang‐Wei Hsieh:** investigation (supporting), writing – original draft (supporting). **Chien‐Wei Feng:** formal analysis (supporting), writing – original draft (supporting). **Zhi‐Cheng Chen:** investigation (supporting), writing – original draft (supporting). **Nan‐Fu Chen:** investigation (supporting), writing – original draft (supporting). **Yung‐Kuo Lee:** investigation (supporting), writing – original draft (supporting). **Tian‐Huei Chu:** conceptualization (equal), formal analysis (equal), investigation (equal), methodology (equal), writing – original draft (equal), writing – review and editing (equal). **Cheng‐Chieh Fang:** conceptualization (equal), formal analysis (equal), investigation (equal), writing – original draft (equal), writing – review and editing (equal). **Nan‐Chieh Huang:** conceptualization (equal), formal analysis (equal), investigation (equal), methodology (equal), writing – original draft (equal), writing – review and editing (equal).

## Conflicts of Interest

The authors declare no conflicts of interest.

## Supporting information


**Appendix S1:** fsn371332‐sup‐0001‐AppendixS1.doc.

## Data Availability

The data are available from the authors upon reasonable request.
