# Supplementary material for: Artificial Intelligence‐Based In Silico Evaluation of the Pharmacological Potential and In Vitro Anti‐Malignancy Effect in the Human Glioblastoma Cell Line of the Hydrate of the Coumarin Compound Meranzin Originating From the Edible Macroalga (Bangia fuscopurpurea)
Source: Food Sci Nutr. 2025 Dec 29;14(1):e71332. doi: 10.1002/fsn3.71332 (PMC12745912; doi:10.1002/fsn3.71332)
Supplement: Supplementary file 1 — Appendix S1: fsn371332‐sup‐0001‐AppendixS1.doc. [file FSN3-14-e71332-s001.doc]

**Supplementary Results**

**Article**

**Artificial intelligence-based *in silico* evaluation of the pharmacological potential and *in vitro* anti-malignancy effect in the human glioblastoma cell line of the hydrate of the coumarin compound meranzin originating from the edible macroalga (*Bangia fuscopurpurea*)**

**Shi-Ying Huang^#^, Yi-Chen Chang^#^, Charles Chien-Chih Chiu, Chang-Wei Hsieh, Chien-Wei Feng, Zhi-Cheng Chen, Nan-Fu Chen, Yung-Kuo Lee, Tian-Huei Chu^*^, Cheng-Chieh Fang^*^, Nan-Chieh Huang^*^**

**# These authors contributed equally to this work.**

**^*^ Corresponding authors.**

**Meranzin**

**Meranzin hydrate**


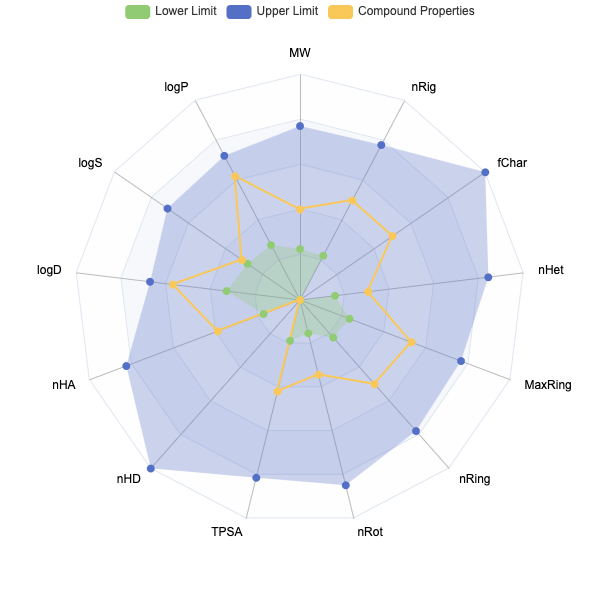

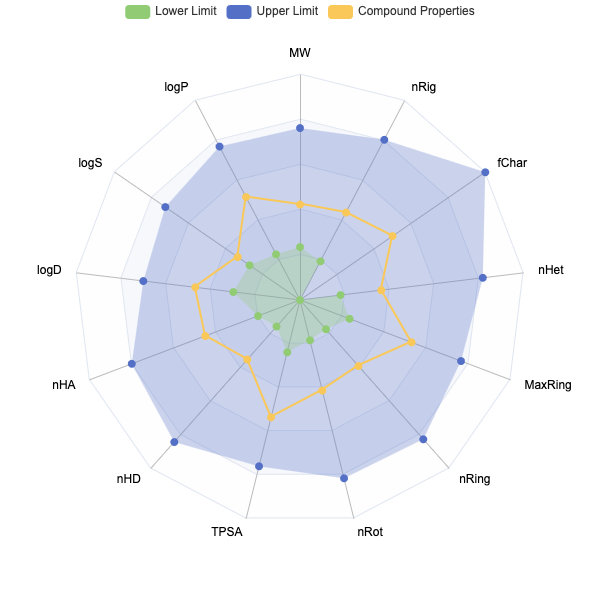


**Supplementary Figure S1. Radar charts of the predicted physicochemical properties of meranzin and its hydrate.** The radar charts display the physicochemical properties of each test compound (in yellow) and the reference optimal ranges (in blue and green), as provided by ADMETlab 3.0.


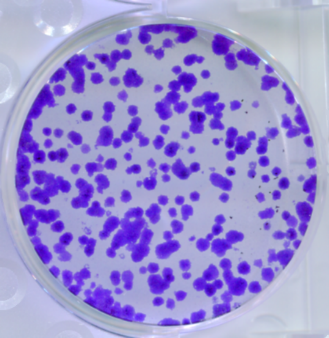

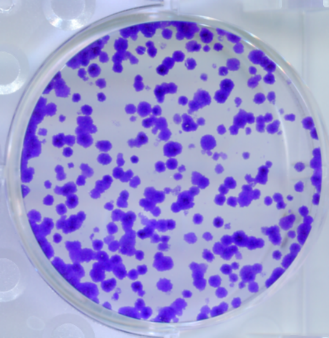

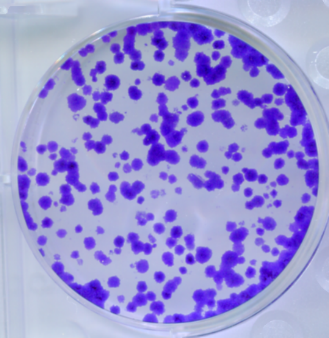

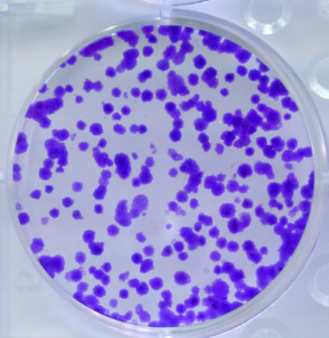

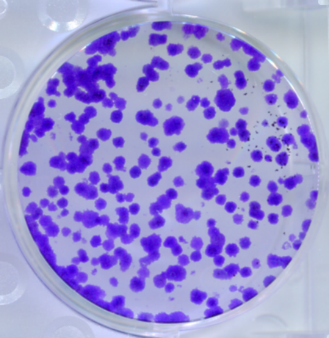

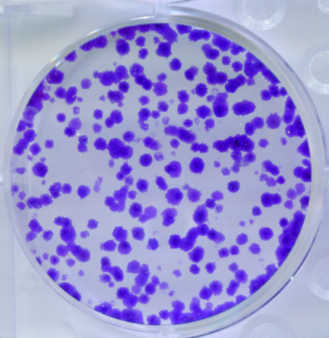


0

0.5

1

5

10

50

Meranzin (μM)

GBM8401

**Supplementary Figure S2. Effect of meranzin hydrate (Meranzin) on** **colony formation in GBM8401 cells.** After 7 days of treatment, meranzin hydrate (0.5–50 μM) did not significantly affect colony formation in GBM8401 cells relative to the control (vehicle) group (*n* ＝ 3).

**Supplementary Table S1. Enrichment analysis of the predicted effects of meranzin and its hydrate on proteins associated with GBM in the DisGeNET database.**

| **I. Downregulation model (*Pa* ＞ *Pi*)** | | | | | | | | | | | | | |
| --- | --- | --- | --- | --- | --- | --- | --- | --- | --- | --- | --- | --- | --- |
|  |  | **Meranzin** (752 predicted genes) | | | | | | **Meranzin hydrate** (823 predicted genes) | | | | | |
|  | Name  (ID disease) | n | N | Odds ratio | p-value | adj.p | *adj.p* ＜0.05 | n | N | Odds ratio | p-value | adj.p | *adj.p*＜0.05 |
| 1 | GBM  (C0017636) | 24 | 93 | 8.238 | 1.83e-13 | 9.99e-13 | Yes | 26 | 93 | 8.152 | 2.79e-14 | 1.66e-13 | Yes |
| 2 | GBM Multiforme  (C1621958) | 29 | 115 | 8.098 | 9.28e-16 | 6.32e-15 | Yes | 30 | 115 | 7.638 | 1.40e-15 | 9.33e-15 | Yes |
| 3 | Giant Cell GBM  (C0334588) | 24 | 84 | 9.123 | 2.78e-14 | 1.61e-13 | Yes | 25 | 84 | 8.670 | 2.66e-14 | 1.58e-13 | Yes |
| **II. Upregulation model (*Pa* ＞ *Pi*)** | | | | | | | | | | | | | |
|  |  | **Meranzin** (1062 predicted genes) | | | | | | **Meranzin hydrate** (1121 predicted genes) | | | | | |
|  | Name  (ID disease) | n | N | Odds ratio | p-value | adj.p | *adj.p* ＜0.05 | n | N | Odds ratio | p-value | adj.p | *adj.p* ＜0.05 |
| 1 | GBM  (C0017636) | 29 | 93 | 7.015 | 4.57e-14 | 3.10e-13 | Yes | 31 | 93 | 7.107 | 5.49e-15 | 3.61e-14 | Yes |
| 2 | GBM Multiforme  (C1621958) | 34 | 115 | 6.677 | 1.12e-15 | 8.43e-15 | Yes | 36 | 115 | 6.699 | 1.82e-16 | 1.35e-15 | Yes |
| 3 | Giant Cell GBM  (C0334588) | 27 | 84 | 7.220 | 1.88e-13 | 1.21e-12 | Yes | 29 | 84 | 7.350 | 1.97e-14 | 1.26e-13 | Yes |

n: the number of downregulated (or upregulated) genes predicted for the test compound with *Pa* ＞ *Pi*, associated with the particular disease (e.g., GBM).

N: the number of background genes annotated in the DisGeNET database, associated with the particular disease.

**Supplementary Table S2. Predicted physicochemical properties of meranzin and its hydrate.**

|  | Property | Comment | **Meranzin** | **Meranzin hydrate** |
| --- | --- | --- | --- | --- |
| 1 | Molecular weight (MW) | Contain hydrogen atoms. **Optimal**: 100—600. | 260.1 | 278.12 |
| 2 | Volume | Van der Waals volume. | 264.305 | 281.652 |
| 3 | Density | Density ＝ MW / Volume | 0.984 | 0.987 |
| 4 | nHA | Number of hydrogen bond acceptors. **Optimal**: 0—12. | 4.0 | 5.0 |
| 5 | nHD | Number of hydrogen bond donors. **Optimal**: 0—7. | 0.0 | 2.0 |
| 6 | nRot | Number of rotatable bonds. **Optimal**: 0—11. | 3.0 | 4.0 |
| 7 | nRing | Number of rings. **Optimal**: 0—6. | 3.0 | 2.0 |
| 8 | MaxRing | Number of atoms in the biggest ring. **Optimal**: 0—18. | 10.0 | 10.0 |
| 9 | nHet | Number of heteroatoms. **Optimal**: 1—15. | 4.0 | 5.0 |
| 10 | fChar | Formal charge. **Optimal**: -4—4. | 0.0 | 0.0 |
| 11 | nRig | Number of rigid bonds. **Optimal**: 0—30. | 15.0 | 12.0 |
| 12 | Flexibility | Flexibility ＝ nRot / nRig | 0.2 | 0.333 |
| 13 | Stereo centers | **Optimal**: ≦ 2 | 1.0 | 1.0 |
| 14 | TPSA | Topological polar surface area. **Optimal**: 0—140. | 51.97 | 79.9 |
| 15 | logS | The logarithm of aqueous solubility value. | -3.691 | -3.369 |
| 16 | logP | The logarithm of the n-octanol/water distribution coefficients. | 2.316 | 1.591 |
| 17 | logD7.4 | logP at pH ＝ 7.4. | 2.402 | 1.848 |
| 18 | pka (Acid) |  | 7.789 | 8.712 |
| 19 | pka (Base) |  | 3.42 | 3.793 |
| 20 | Melting point |  | 123.059 | 191.178 |
| 21 | Boiling point |  | 303.625 | 315.548 |

pka (Acid or Base): Acid-base dissociation constant (pKa) value represents the strength of a drug molecule’s acidity or basicity.

Melting point: The predicted melting point of a compound is expressed in degrees Celsius (℃). Melting points below 25 ℃are classified as liquids, while melting points above 25 ℃ are classified as solids.

Boiling point: The predicted boiling point of a compound is expressed in degrees Celsius (℃). A normal boiling point below 25 ℃ is categorized as a gas.

**Supplementary Table S3. Predicted medicinal chemistry properties of meranzin and its hydrate.**

|  | Property | Comment | **Meranzin** | **Meranzin hydrate** |
| --- | --- | --- | --- | --- |
| 1 | QED | A measure of drug-likeness based on the concept of desirability:  **Attractive**: ＞ 0.67; unattractive: 0.49—0.67; too complex: ＜0.34. | 0.628 | 0.828 |
| 2 | SAscore | Synthetic accessibility score is designed to estimate ease of synthesis of drug-like molecules. Difficult: ≧ 6; **easy**: ＜ 6. | Easy  (3.0) | Easy  (3.0) |
| 3 | GASA | The probability of being difficult to synthesize, ranging from 0 to 1. | Easy (0.0) | Easy (0.0) |
| 4 | Fsp^3^ | The number of sp3 hybridized carbons / total carbon count, correlating with melting point and solubility. **Suitable**: ≧ 0.42. | 0.4 | 0.4 |
| 5 | MCE-18 | Medicinal chemistry evolution. **Suitable**: ≧ 45. | 57.429 | 30.0 |
| 6 | NPscore | Natural product-likeness score. Typically: -5—5. The higher the score is, the higher the probability is that the molecule is a Natural product. | 1.816 | 1.65 |
| 7 | Lipinski rule | MW ≦ 500; logP ≦ 5; Hacc ≦ 10; Hdon ≦ 5. If two properties are out of range, a poor absorption or permeability is possible, one is acceptable. | Accepted | Accepted |
| 8 | Pfizer rule | logP ＞ 3; TPSA ＜ 75. Compounds with a high logP (＞ 3) and low TPSA (＜ 75) are likely to be toxic. | Accepted | Accepted |
| 9 | GSK rule | MW ≦ 400; logP ≦ 4. Compounds satisfying the GSK rule may have a more favorable ADMET profile. | Accepted | Accepted |
| 10 | Golden Triangle | 200 ≦ MW ≦ 500; -2 ≦ logD ≦ 5. Compounds satisfying the Golden Triangle rule may have a more favorable ADMET profile. | Accepted | Accepted |
| 11 | PAINS | Frequent hitters, alpha-screen artifacts and reactive compound 480 substructures. | 0 | 0 |
| 12 | Alarm NMR rule | Thiol reactive compounds. | 2 alerts | 2 alerts |
| 13 | BMS rule | Undesirable, reactive compounds 176 substructures. | 0 | 0 |
| 14 | Chelating | Chelating rule: chelating compounds. | 0 | 0 |
| 15 | Colloidal aggregators | The probability of being colloidal aggregators.  Non-colloidal aggregators: 0; colloidal aggregators: 1. | 0.362 | 0.41 |
| 16 | FLuc inhibitors | The probability of being fLuc inhibitors.  Non-fLuc inhibitors: 0; fLuc inhibitors: 1. | 0.306 | 0.088 |
| 17 | Blue fluorescence | The probability of being blue fluorescence.  Non-blue fluorescence: 0; blue fluorescence: 1. | 0.98 | 0.993 |
| 18 | Green fluorescence | The probability of being green fluorescence.  Non-green fluorescence: 0; green fluorescence: 1. | 0.419 | 0.133 |
| 19 | Reactive compounds | The probability of being reactive compounds.  Non-reactive compound: 0; reactive compound: 1. | 0.446 | 0.16 |
| 20 | Promiscuous compounds | The probability of being promiscuous compounds.  Non-promiscuous compound: 0; promiscuous compound: 1. | 0.642 | 0.297 |

**Supplementary Table S4. Predicted absorption and distribution of meranzin and its hydrate.**

|  | Property | Comment | **Meranzin** | **Meranzin hydrate** |
| --- | --- | --- | --- | --- |
| **I. Absorption** | | | | |
| 1 | Caco-2 permeability | **Optimal**: higher than -5.15 Log unit. | -4.471 | -4.635 |
| 2 | MDCK permeability | Low permeability: ＜ 2 × 10^-6^ cm/s; medium permeability: 2—20 × 10^-6^ cm/s; high passive permeability: ＞ 20 × 10^-6^ cm/s | -4.579 | -4.5 |
| 3 | PAMPA | The experimental data for Peff was logarithmically transformed (logPeff).  Low-permeability: logPeff values below 2.0: 0; High-permeability: logPeff values exceeding 2.5: 1. | --  (0.256) | ++  (0.819) |
| 4 | Pgp inhibitor | The probability of being inhibitor. Non-inhibitor: 0; inhibitior: 1. | --- (0.047) | --- (0.0) |
| 5 | Pgp substrate | The probability of being substrate. Non-substrate: 0; substrate: 1. | --- (0.0) | --- (0.001) |
| 6 | HIA | Human intestinal absorption (HIA). The probability of being HIA＋.  HIA－ (HIA ≧ 30 %): 0; HIA＋ (HIA ＜ 30 %): 1. | ---  (0.005) | ---  (0.0) |
| 7 | F20% | 20 % bioavailability (BAB). The probability of being F20%＋.  F20%－ (BAB ≧ 20 %): 0; F20%＋ (BAB ＜ 20 %): 1. | ++  (0.783) | --  (0.104) |
| 8 | F30% | 30 % bioavailability (BAB). The probability of being F30%＋.  F30%－ (BAB ≧ 30 %): 0; F30%＋ (BAB ＜ 30 %): 1. | -  (0.47) | ---  (0.036) |
| 9 | F50% | 50 % bioavailability (BAB). The probability of being F50%＋.  F50%－ (BAB ≧ 50 %): 0; F50%＋ (BAB ＜ 50 %): 1. | +  (0.57) | --  (0.19) |
| **II. Distributions** | | | | |
| 1 | PPB | Plasma protein binding. **Optimal**: ＜ 90 %. Drugs with high protein-bound may have a low therapeutic index. | 84.439 % | 69.147 % |
| 2 | logVDss | Volume Distribution. **Optimal**: 0.04—20 L/Kg. | 0.094 | -0.118 |
| 3 | BBB | Blood-brain barrier penetration (BBB). The probability of being BBB＋.  BBB－: 0 ; BBB＋: 1. | ---  (0.004) | ---  (0.0) |
| 4 | Fu | The fraction unbound in plasmas. Low: ＜ 5%; middle: 5—20 %; high: ＞ 20 %. | 14.549 % | 26.992 % |
| 5 | OATP1B1 inhibitor | The probability of being inhibitor. Non-inhibitor: 0; inhibitor: 1. | +++  (0.996) | +++  (0.994) |
| 6 | OATP1B3 inhibitor | The probability of being inhibitor. Non-inhibitor: 0; inhibitor: 1. | +++  (1.0) | +++  (1.0) |
| 7 | BCRP inhibitor | The probability of being inhibitor. Non-inhibitor: 0; inhibitor: 1. | ---  (0.001) | ---  (0.001) |
| 8 | MRP1 inhibitor | The probability of being inhibitor. Non-inhibitor: 0; inhibitor: 1. | +++  (0.992) | +++  (0.983) |
| 9 | BSEP inhibitor | The probability of being inhibitor. Non-inhibitor: 0; inhibitor: 1. | +++  (0.995) | ++  (0.899) |

For the classification endpoints, the prediction probability values are transformed into six symbols: 0—0.1 (---), 0.1—0.3 (--), 0.3—0.5 (-), 0.5—0.7 (+), 0.7—0.9 (++), and 0.9—1.0 (+++).

Peff: effective intestinal membrane permeability.

**Supplementary Table S5. Predicted metabolism and excretion of meranzin and its hydrate.**

|  | Property | Comment | **Meranzin** | **Meranzin hydrate** |
| --- | --- | --- | --- | --- |
| **I. Metabolism** | | | | |
| 1 | CYP1A2 inhibitor | The probability of being inhibitor. Non-inhibitor: 0; inhibitor: 1. | +++  (0.999) | +  (0.661) |
| 2 | CYP1A2 substrate | The probability of being substrate. Non-substrate: 0; substrate: 1. | +++  (0.901) | ---  (0.0) |
| 3 | CYP2C19 inhibitor | The probability of being inhibitor. Non-inhibitor: 0; inhibitor: 1. | -  (0.309) | ++  (0.778) |
| 4 | CYP2C19 substrate | The probability of being substrate. Non-substrate: 0; substrate: 1. | ++  (0.75) | ---  (0.0) |
| 5 | CYP2C9 inhibitor | The probability of being inhibitor. Non-inhibitor: 0; inhibitor: 1. | --  (0.137) | ---  (0.005) |
| 6 | CYP2C9 substrate | The probability of being substrate. Non-substrate: 0; substrate: 1. | -  (0.436) | --  (0.297) |
| 7 | CYP2D6 inhibitor | The probability of being inhibitor. Non-inhibitor: 0; inhibitor: 1. | ---  (0.074) | ---  (0.0) |
| 8 | CYP2D6 substrate | The probability of being substrate. Non-substrate: 0; substrate: 1. | ---  (0.001) | +++  (0.973) |
| 9 | CYP3A4 inhibitor | The probability of being inhibitor. Non-inhibitor: 0; inhibitor: 1. | +++  (0.92) | ++  (0.836) |
| 10 | CYP3A4 substrate | The probability of being substrate. Non-substrate: 0; substrate: 1. | +++  (0.996) | ---  (0.02) |
| 11 | CYP2B6 inhibitor | The probability of being inhibitor. Non-inhibitor: 0; inhibitor: 1. | +++  (0.997) | ++  (0.854) |
| 12 | CYP2B6 substrate | The probability of being substrate. Non-substrate: 0; substrate: 1. | -  (0.492) | ---  (0.0) |
| 13 | CYP2C8 inhibitor | The probability of being inhibitor. Non-inhibitor: 0; inhibitor: 1. | +++  (0.973) | --  (0.157) |
| 14 | HLM stability | Human liver microsomal (HLM) stability. The probability of HLM instability. Stable (HLM ＞ 30 min): 0 ; unstable (HLM ≦ 30 min): 1. | +++  (0.968) | +++  (0.956) |
| **II. Excretions** | | | | |
| 1 | CL_plasma_ | The unit of predicted CL_plsama_ penetration is mL/min/Kg.  High clearance: ＞ 15; moderate clearance: 5—15; low clearance: ＜ 5. | 8.637 | 9.202 |
| 2 | T1/2 | The unit of predicted T1/2 is hours.  Ultra-short half-life drugs: ＜ 1; short half-life drugs: 1—4;  intermediate short half-life drugs: 4—8; long half-life drugs: ＞ 8. | 0.578 | 0.951 |

For the classification endpoints , the prediction probability values are transformed into six symbols: 0—0.1 (---), 0.1—0.3 (--), 0.3—0.5 (-), 0.5—0.7 (+), 0.7—0.9 (++), and 0.9—1.0 (+++).

1. **Supplementary Table S6. Predicted results based on toxicophore rules for meranzin and its hydrate.**

|  | Property | Comment | **Meranzin** | **Meranzin hydrate** |
| --- | --- | --- | --- | --- |
| 1 | Aquatic toxicity rule | 99 substructures: toxicity to liquid (water). | 2 alerts | 2 alerts |
| 2 | Genotoxic carcinogenicity mutagenicity rule | 117 substructures: carcinogenicity or mutagenicity. | 6 alerts | 1 alert |
| 3 | Nongenotoxic carcinogenicity rule | 23 substructures: carcinogenicity through nongenotoxic mechanisms. | 1 alert | 0 |
| 4 | Skin sensitization rule | 155 substructures: skin irritation. | 3 alerts | 0 |
| 5 | Acute toxicity rule | 20 substructures: acute toxicity during oral administration. | 0 | 0 |
| 6 | Non-biodegradable rule | 19 substructures: non-biodegradable. | 2 alerts | 0 |
| 7 | SureChEMBL rule | 164 substructures: MedChem unfriendly status. | 2 alerts | 0 |
| 8 | FAF-Drugs4 rule | 154 toxic substructures from FAF-Drug4 | 2 alerts | 1 alert |

**Supplementary Table S7. Predicted toxicity and environmental toxicity of meranzin and its hydrate.**

|  | Property | Comment | **Meranzin** | **Meranzin hydrate** |
| --- | --- | --- | --- | --- |
| **I. Toxicities** | | | | |
| 1 | hERG blockers | The probability of being blockers.  IC_50_ ＞ 10 μM or ＜ 50 % inhibition at 10 μM: 0;  IC_50_ ≦ 10 μM or ≧ 50 % inhibition at 10 μM: 1. | 0.116 | 0.121 |
| 2 | hERG blockers (10 μM) | The probability of being blockers.  IC_50_ ＞ 10 μM: 0; IC_50_ ≦ 10 μM: 1. | 0.375 | 0.338 |
| 3 | DILI | Drug induced liver injury (DILI). The probability of being toxic.  No risk: 0; high risk: 1. | 0.893 | 0.285 |
| 4 | Ames toxicity | The probability of being toxic. Negative: 0; positive: 1. | 0.85 | 0.574 |
| 5 | Rat oral acute toxicity | The probability of being toxic.  Low toxicity (＞ 500 mg/Kg): 0; high toxicity (＜ 500 mg/Kg): 1. | 0.585 | 0.376 |
| 6 | FDAMDD | FDA maximum (recommended) daily dose (FDAMDD). The probability of being positive. Negative: 0; positive: 1. | 0.627 | 0.694 |
| 7 | Skin sensitization | The probability of being toxic. Non-sensitizer: 0; sensitizer: 1. | 0.738 | 0.251 |
| 8 | Carcinogenicity | The probability of being toxic. Non-carcinogens: 0; carcinogens: 1. | 0.79 | 0.616 |
| 9 | Eye corrosion | The probability of being corrosives. Negative: 0; positive: 1. | 0.326 | 0.016 |
| 10 | Eye irritation | The probability of being irritants. Negative: 0; positive: 1. | 0.974 | 0.731 |
| 11 | Respiratory | The probability of being respiratory toxicants. Negative: 0; positive: 1. | 0.671 | 0.702 |
| 12 | Human hepatotoxicity | The probability of being toxic. Negative: 0; positive: 1. | 0.622 | 0.471 |
| 13 | Drug-induced nephrotoxicity | The probability of being toxic. Negative: 0; positive: 1. | 0.425 | 0.296 |
| 14 | Drug-induced neurotoxicity | The probability of being toxic. Negative: 0; positive: 1. | 0.684 | 0.392 |
| 15 | Ototoxicity | The probability of being toxic. Negative: 0; positive: 1. | 0.226 | 0.328 |
| 16 | Hematotoxicity | The probability of being toxic. Negative: 0; positive: 1. | 0.401 | 0.191 |
| 17 | Genotoxicity | The probability of being toxic. Negative: 0; positive: 1. | 0.966 | 0.778 |
| 18 | RPMI-8226 immunitoxicity | The probability of being toxic. Negative: 0; positive: 1. | 0.111 | 0.066 |
| 19 | A549 cytotoxicity | The probability of being toxic. Negative: 0; positive: 1. | 0.125 | 0.079 |
| 20 | Hek293 cytotoxicity | The probability of being toxic. Negative: 0; positive: 1. | 0.341 | 0.321 |
| **II. Environmental toxicities** | | | | |
| 1 | BCF | Bioconcentration factors are used for considering secondary poisoning potential and assessing risks to human health via the food chain.  The unit is －log10[(mg/L)/(1000 × MW)]. | 1.437 | 0.355 |
| 2 | IGC_50_ | Tetrahymena pyriformis 50 percent growth inhibition concentration.  The unit is －log10[(mg/L)/(1000 × MW)]. | 4.052 | 3.025 |
| 3 | LC_50_DM | 48-hour daphnia magna 50 percent lethal concentration.  The unit is －log10[(mg/L)/(1000 × MW)]. | 5.291 | 4.289 |
| 4 | LC_50_FM | 96-hour fathead minnow 50 percent lethal concentration..  The unit is －log10[(mg/L)/(1000 × MW)]. | 4.923 | 3.585 |

**Supplementary Table S8. Predicted possible effects on Tox21 pathways for meranzin and its hydrate.**

|  | Property | Comment | **Meranzin** | **Meranzin hydrate** |
| --- | --- | --- | --- | --- |
| 1 | NR-AhR | Aryl hydrocarbon receptor. The probability of being active.  Inactives: 0; actives: 1. | +  (0.62) | -  (0.499) |
| 2 | NR-AR | Androgen receptor. The probability of being active.  Inactives: 0; actives: 1. | +  (0.512) | --  (0.111) |
| 3 | NR-AR-LBD | Androgen receptor ligand-binding domain.  The probability of being active. Inactives: 0; actives: 1. | ---  (0.063) | ---  (0.005) |
| 4 | NR-Aromatase | The probability of being active. Inactives: 0; actives: 1. | +  (0.645) | ---  (0.028) |
| 5 | NR-ER | Estrogen receptor.  The probability of being active. Inactives: 0; actives: 1. | -  (0.358) | -  (0.361) |
| 6 | NR-ER-LBD | Estrogen receptor ligand-binding domain.  The probability of being active. Inactives: 0; actives: 1. | --  (0.265) | ---  (0.018) |
| 7 | NR-PPAR-gamma | Peroxisome proliferator-activated receptor gamma.  The probability of being active. Inactives: 0; actives: 1. | ---  (0.095) | ---  (0.016) |
| 8 | SR-ARE | Antioxidant response element.  The probability of being active. Inactives: 0; actives: 1. | -  (0.433) | ---  (0.058) |
| 9 | SR-ATAD5 | ATPase family AAA domain-containing protein 5.  The probability of being active. Inactives: 0; actives: 1. | -  (0.375) | ---  (0.003) |
| 10 | SR-HSE | Heat shock factor response element.  The probability of being active. Inactives: 0; actives: 1. | ++  (0.705) | ---  (0.053) |
| 11 | SR-MMP | Mitochondrial membrane potential.  The probability of being active. Inactives: 0; actives: 1. | ++  (0.728) | -  (0.363) |
| 12 | SR-p53 | p53, a tumor suppressor protein.  The probability of being active. Inactives: 0; actives: 1. | +  (0.647) | ---  (0.042) |

For the classification endpoints , the prediction probability values are transformed into six symbols: 0—0.1 (---), 0.1—0.3 (--), 0.3—0.5 (-), 0.5—0.7 (+), 0.7—0.9 (++), and 0.9—1.0 (+++).
